# Reliability and Validity of the Thai Version of the Florida Obsessive-Compulsive Inventory

**DOI:** 10.1155/2015/240787

**Published:** 2015-03-15

**Authors:** Ratana Saipanish, Thanita Hiranyatheb, Manote Lotrakul

**Affiliations:** Department of Psychiatry, Ramathibodi Hospital, Faculty of Medicine, Mahidol University, Rama VI Road, Rajthevi, Bangkok 10400, Thailand

## Abstract

This study aimed to examine the reliability and validity of the Thai version of the FOCI (FOCI-T), which is a brief self-report questionnaire to assess the symptoms and severity of obsessive-compulsive disorder (OCD). Forty-seven OCD patients completed the FOCI-T, the Patient Health Questionnaire (PHQ-9), and the Pictorial Thai Quality of Life (PTQL). They were then interviewed to determine the OCD symptom severity by the Yale-Brown Obsessive-Compulsive Scale-Second Edition (YBOCS-II) and depressive symptoms by the Hamilton Rating Scale for Depression (HAM-D), together with the Global Assessment of Functioning (GAF) and the Clinical Global Impression-Severity Scales (CGI-S). The result showed that the FOCI-T had satisfactory internal consistency reliability on both the Symptom Checklist (KR-20 = 0.86) and the Severity Scale (*α* = 0.92). Regarding validity analyses, the FOCI-T Severity Scale had stronger correlations with the YBOCS-II and CGI-S than the FOCI-T Symptom Checklist. This implied the independence between the FOCI-T Symptom Checklist and the Severity Scale and good concurrent validity of the FOCI-T Severity Scale. Our results suggested that the FOCI-T was found to be a reliable and valid self-report measure to assess obsessive-compulsive symptoms and severity.

## 1. Introduction

Although the Yale-Brown Obsessive-Compulsive Scale (YBOCS; 1989) [1] is well-known as a gold standard measure for the symptom severity of Obsessive-Compulsive Disorder (OCD), it is a clinician-administered instrument which might take time in most cases. Therefore, self-report measurement might be another option for the clinicians who are facing a high patient load in their daily practice.

A number of self-report measures were developed to assess obsessions and/or compulsions and their severity, such as the Maudsley Obsessional-Compulsive Inventory (MOCI) [2], the Compulsive Activity Checklist [3], the Padua Inventory Revised [4], the Obsessive-Compulsive Inventory-Revised (OCI-R) [5], the Vancouver Obsessional Compulsive Inventory (VOCI) [6], and the Dimensional Obsessive-Compulsive Scale [7]. All of them, including the Yale-Brown Obsessive-Compulsive Scale-Self Report (YBOCS-SR) [8], are limited in their ability to assess rapidly both OCD symptoms and overall severity. A brief assessment tool is always needed, especially in crowded clinical settings. From the aforementioned reasons, Goodman [9] developed the Florida Obsessive-Compulsive Inventory (FOCI) and Storch et al. [10] initially validated this instrument in 2007. Its major strength is that it is a self-report questionnaire, which provides a quick evaluation for both OCD symptoms and their severity.

Thus far only a few studies have evaluated the reliability and validity of the FOCI [10–12], although it is a very useful measure for assessing OCD. It is considered that the FOCI would be suitable for many countries in which mental health care service problems arise from the disparity between high number of patients and small number of psychiatrists, especially Thailand [13]. Although the Thai version of YBOCS-Second Edition is used as a measurement tool for OCD [14] in Thailand, it remains a clinician-rated and time-consuming measure. Therefore, this study aimed to evaluate the reliability and validity of the Thai Version of the Florida Obsessive-Compulsive Inventory (FOCI-T) to assess symptoms and severity of the OCD in clinical samples.

## 2. Methods 

### 2.1. Subjects and Procedure

This study protocol was approved by the Ethics Committee of the Faculty of Medicine, Ramathibodi Hospital, Bangkok. All participants provided their written informed consent before participating in this study; the parents of two participants aged under 20 provided their informed consent as witnesses.

Participants included patients who were between 17 and 70 years of age and were diagnosed as individuals with OCD symptoms by trained psychiatrists at the out-patient clinic of the Department of Psychiatry, Ramathibodi Hospital, Bangkok. The diagnosis of OCD was defined by the Diagnostic and Statistical manual of Mental Disorders, fourth edition, text revision (DSM-IV-TR), and was confirmed by the use of the Mini International Neuropsychiatric Interview (MINI) [15], a structured diagnostic psychiatric interview in the Thai version [16]. This was administered by a research assistant, a clinical psychologist, who was trained to use this instrument. Exclusion criteria included illiteracy, mental retardation, active psychosis, active mood disorders, or organic mental syndrome.

With regard to inclusion and exclusion criteria, eighty-one OCD patients were invited to participate in this study. After explaining the objectives and the process of the study, a total of 49 OCD patients agreed to participate. After the informed consents were obtained, all subjects completed the FOCI-T, the Patient Health Questionnaire (PHQ-9), and the Pictorial Thai Quality of Life (PTQL), followed by an interview by one of our four interviewers. The interview process took 90 to 120 minutes. This included the assessment of the OCD symptoms by the Thai version of YBOCS-Second Edition (YBOCS-II) and the assessment of depression by the Hamilton Rating Scale for Depression (HAM-D). Finally, the third author (ML), an experienced psychiatrist who was blind to all aforementioned measurement scores, assessed the patients' functioning according to the Global Assessment of Functioning scale (GAF) and the symptom severity according to the Clinical Global Impression rating scales (CGI-S).

Four interviewers, two psychiatrists and two psychiatric nurses, were adequately trained to use the Thai version of YBOCS-II and HAM-D by attending didactic seminars, observing a VDO of patients being interviewed, practicing to administer the measures and discussing the discrepancies between raters. Our interrater reliability was 0.97 for the HAM-D, and 0.99 for the YBOCS-II [14].

## 3. Measures 

### 3.1. FOCI-T

The FOCI comprises two scales: the Symptom Checklist and the Symptom Severity, which can be completed in 5 minutes. On the Symptom Checklist scale, the patient would mark the presence (“yes” = 1) or absence (“no” = 0) of common obsessions (10 items) and compulsions (10 items) that were developed from the YBOCS-Symptom Checklist. The total score of the Symptom Checklist is calculated by summing up the presence of all items (range = 0–20), with higher scores corresponding to a greater number of symptoms. On the Symptom Severity scale, the patient would rate the severity level (from 0 to 4) of endorsed symptoms on five items: time occupied; distress; degree of control; avoidance; and life interference. The total severity score is calculated by summing up the five severity items (range = 0–20), with higher scores corresponding to a greater symptom severity.

The English version of the FOCI demonstrated excellent psychometric properties in assessing the presence and severity of obsessive-compulsive symptoms [10]. It showed strong internal consistency for both the Symptom Checklist (Kuder-Richardson-20 (KR-20) = 0.83) and the Severity Scale (Cronbach's alpha (*α*) = 0.89). Concurrent validity was reported by strong correlations between the FOCI Severity Scale and the clinician-rated measures of OCD symptom severity (*r*s > 0.8) and moderate to strong correlations with measures of functioning impairment and depressive symptoms.

After obtaining permission from the copyright holder, the researchers proceeded to translate the FOCI into Thai language. The process included three independent forward translations, making consensus between the translators on a forward translation, backward-translation by a bilingual English teacher, cross-cultural adaptation, and pilot testing by ten invited out-patients. Finally, the last adjusted version of FOCI-T was completed.

### 3.2. Yale-Brown Obsessive-Compulsive Scale-Second Edition (YBOCS-II)

The YBOCS-II [17] was developed through revision in order to improve some areas of the original version of the YBOCS, which is a semistructured, clinician-administered measure that has been widely considered for two decades to be the gold standard measure of obsessive-compulsive severity. In the YBOCS-II, severity items are rated over the previous week on a 6-point response scale ranging from 0 to 5. The first five items assess the severity of obsession; the other five items assess compulsion. All ten items are summed up to derive the Obsession and Compulsion Severity Scale scores which range from 0 to 50, with a higher score corresponding to a higher symptom severity. The YBOCS-II English version has excellent psychometric properties in assessing the presence and severity of obsessive-compulsive symptoms. The Thai version of YBOCS-II [14] also showed strong internal consistency for the Symptom Checklist (KR-20 = 0.90) and the severity scale (*α* = 0.94). The factor analysis of the Severity Scale revealed two factors generally consistent with the obsession and compulsion subscales. Construct validity was also reported strong correlations with clinician-rated measures of the symptom severity and moderate correlations with measures of the depressive symptoms.

### 3.3. Hamilton Rating Scale for Depression (HAM-D)

The HAM-D [18] is a widely accepted research measure to assess the severity of depression and response to treatment. Its score has to be rated by healthcare professional. A score of 0–7 is generally accepted to be within normal range, while a higher score is considered to be clinical depression; the higher the score, the more severe the depression. The Thai version of the HAM-D shows adequate internal consistency (*α* = 0.74); its concurrent validity as compared with the Global Assessment Scale is also considered satisfactory (Spearman's correlation coefficient = −0.82) [19].

### 3.4. Patient Health Questionnaire (PHQ-9)

The PHQ-9 [20] is a self-report measure of depression severity, consisting of nine questions based on the nine DSM-IV criteria for major depressive episodes. The patients would rate the level of the symptoms that they experienced during the two weeks prior to answering the questionnaire. Scores of each item range from 0 (not at all) to 3 (nearly every day), and accumulated scores range from 0 to 27. The Thai version of the PHQ-9 shows satisfactory internal consistency (*α* = 0.79) and moderate convergent validity as compared with the HAM-D (*r* = 0.56) [21].

### 3.5. Clinical Global Impression-Severity Scales (CGI-S)

The CGI-S [22] is commonly used to measure the symptom severity of patients with mental disorders. It is a 7-point clinician rating scale: 1 = normal, not at all ill; 2 = borderline mentally ill; 3 = mildly ill; 4 = moderately ill; 5 = markedly ill; 6 = severely ill; or 7 = extremely ill. The clinicians would rate the scale which is based upon how is the nature of the patients' symptoms.

### 3.6. The Global Assessment of Functioning (GAF)

The GAF [23] is a worldwide scoring system used by the mental health clinicians to subjectively rate the psychological, social, and occupational functioning of how adaptively a psychiatric patient truly is. It covers the range from severe psychopathology to positive mental health. The score ranges from 0 to 100. The higher the score of the patient is, the higher the function he/she has.

### 3.7. The Pictorial Thai Quality of Life (PTQL)

The PTQL is a self-report tool to measure mental illness both in a clinical and a Thai community setting. It consists of 25 items divided into six domains: Physical, Cognitive, Affective, Social Function, Economic, and Self-Esteem. The score ranges from 0 to 72, 0–24 = poor; 25–49 = average; 50–72 = good quality of life. All items possess sufficient discriminant power. It was found to be statistically significant different (*P* ≤ 0.001) between those people with and without mental disorders. It showed a high level of concurrent validity association with WHOQOL-BREF (*r* = 0.92). Additionally, the internal consistency reliability of the PTQL was excellent (*α* = 0.88) [24].

### 3.8. Data Analysis

The Statistical Package for the Social Science 18.0 (SPSS 18.0) was used for data analysis. Each of the FOCI Symptom Checklist items was reported in frequency, and each of the FOCI Severity Scale items was reported in both the mean and standard deviation. In terms of reliability analysis, the internal consistency was assessed with the Kuder-Richardson-20 (KR-20) statistic for the Symptom Checklist and Cronbach's alpha (*α*) for the Severity Scale. Both the KR-20 reliability index and alpha coefficient of a magnitude of ≥0.7 were considered to be an acceptable value of reliability [25]. Concerning the validity analysis, the construct validity was assessed by performing an exploratory factor analysis with principle component extraction and varimax rotation. The Kaiser-Meyer-Olkin (KMO) and Bartlett tests were performed to assess the sampling adequacy and sphericity of the data. Furthermore, the concurrent validity was evaluated by assessing the Pearson product-moment correlations between the FOCI and other measures.

## 4. Results

Out of consented 49 OCD patients, a total of 47 patients participated in the whole process of the study and completed all data. The mean age of the subjects was 37.9 (SD = 16.3). There were 20 females (42.6%) and 27 males (57.4%); most of them (55.3%) were single and half of them graduated from university. According to the Mini-International Neuropsychiatric Interview, 25 patients (53.2%) had other comorbid disorders, with the most prevalent being generalized anxiety disorder and life-time psychosis (14.9%), followed by dysthymia, panic disorder, and social phobia (12.8%).

The FOCI-T items responded to by our samples are listed in Table 1, which shows the frequencies of “yes” responses for the 20 Symptom Checklist items, together with both the mean and standard deviations for the Severity Scale. Participants' scores on the FOCI Symptom Checklist ranged from a minimum of 0 to a maximum of 18; on the Severity Scale ranged from 0 to 19, as shown in Table 2. There were no gender differences for both the mean scores of the FOCI Symptom Checklist and the Severity Scale. The minimum, maximum, mean scores, and standard deviations on the measures of depression (HAM-D and PHQ-9), as well as functioning (GAF), and symptom severity (CGI-S) of the participants are all shown in Table 2.

### 4.1. Reliability

Internal consistency for the FOCI Symptom Checklist was 0.86 as measured by the Kuder-Richardson 20 formula, and Cronbach's alpha coefficient for the FOCI Severity Scale was acceptable (*α* = 0.92). The item-total correlations between each item of the FOCI severity scale are presented in Table 1. All items, if deleted, would consistently decrease the total scale's alpha value.

### 4.2. Factor Analysis

Since the FOCI-T Symptom Checklist, consisting of dichotomous variables (yes/no response), provides the number of common OCD symptoms which were developed from the YBOCS-Symptom Checklist. This part would be very useful for clinicians to provide the comprehensive treatment for all existing symptoms in patients. The factor analysis was therefore examined only for the FOCI-T Severity Scale. Five items of the FOCI-T Severity Scale were analyzed by principle component extraction and varimax rotation techniques. The Kaiser-Meyer-Olkin test of sampling adequacy (0.84) revealed that factor analysis could be used with our data. Bartlett's test was applied to calculate sampling sphericity and demonstrated a high degree of significance (*χ*_(df=10)_^2^ = 167.39,  *P* < 0.0001). The result showed only one factor which explained the 75.2% of variance (eigenvalue >1.0). Table 1 shows the loading factors of each item which ranges from 0.81–0.95.

### 4.3. Validity

To determine convergent and divergent validity, Table 2 presents the correlations between the FOCI-T Symptom Checklist, the FOCI-T Severity Scale, and other measures assessing OCD symptom severity, functioning impairment, depression, and quality of life. Since all measures had different levels of internal consistency reliability in this sample, the correlations were corrected for attenuation due to measurement errors. The correction for attenuation (*R*_*xy*_ = *r*_*xy*_/sqrt(*r*_*xx*_ *r*_*yy*_)) indicates what the true correlation would be if the perfect reliability was measured. However, correlations involving the CGI-S and the GAF were not corrected because no reliability for these two measures was collected. Actual correlations are shown in the lower part of Table 2, and corrected correlations are shown in the upper part (italic font), with reliabilities in the diagonal direction (bold).

The FOCI-T Symptom Checklist showed moderate correlations (*r*s ≤ 0.61) with the FOCI-T Severity Scale, the YBOCS-II obsession, compulsion, total scale, the CGI-S, and all measures of the depression severity; functioning; and quality of life. Having corrected correlation of <0.57 with the severity scales of YBOCS-II, the FOCI-T Symptom Checklist was considered to be insensitive to the severity of OCD symptoms.

At the same time, the FOCI-T Severity Scale was very strong correlated with the YBOCS-II obsession, compulsion, and total score (*r*s > 0.9). It also strongly correlated with the CGI-S (*r* = 0.76), which is an assessment measure for general symptom severity in psychiatric patients. These findings indicated strong convergent validity of the FOCI-T Severity Scale. Divergent validity of the FOCI-T Severity Scale was evidenced by moderate correlation with the severity of depression measured by the HAM-D (*r* = 0.41) and PHQ-9 (*r* = 0.69), moderate negative correlation with the functioning impairment measured by the GAF (*r* = −0.64), and the quality of life measured by the P-TQOL (*r* = −0.57). Having different findings in correlations to the YBOCS-II subscales, the FOCI-T Severity Scale could be implied to be independent from the FOCI-T Symptom Checklist.

## 5. Discussion

This study aimed to assess the reliability and validity of the FOCI-T, which is the Thai version of the OCD self-rated measure. Our results indicated that the FOCI-T is a psychometrically reliable and valid measure for the assessment of symptoms and their severity in OCD patients. The levels of internal consistency of the FOCI-T Symptom Checklist (KR20 = 0.86) and Severity Scale (*α* = 0.92) were both in a good range and similar to the English version of the FOCI by Storch et al. [10], which were 0.83 and 0.89, respectively. A lower level of intercorrelation between the Symptom Checklist and Severity Scale (*r* = 0.60) suggested a relative independence between the number of symptoms and the overall symptom severity.

In support of the measure's convergent validity, the data showed strong correlations between the FOCI-T Severity Scale and the YBOCS-II Severity Scale and CGI-S. The magnitude of correlation between the FOCI-T Severity and the YBOCS-II Severity Scale (*r* = 0.90)was somewhat stronger than the correlation between the FOCI and the YBOCS (*r* = 0.78) studied by Storch et al. [10]. This result might be due to the different versions of YBOCS. In this study the Thai version of the YBOCS-II [14] was used; the YBOCS-II version was developed to improve certain inherent limitations of the original YBOCS. Divergent validity for the FOCI-T Severity Scale was reported through weaker correlations with measures of the severity of depression, quality of life, and functioning. The FOCI-T Severity Score was weakly to moderately correlated with the total severity scores of depression measured by the HAM-D and PHQ-9, which might suggest the presence of high comorbid depression in OCD patients as reported by Aldea et al. [11] and Pinto et al. [26]. In addition, the FOCI-T Severity Score pointed to a moderate correlation with the PTQL and the GAF, which suggested some impairment in the quality of life and functioning of the OCD patients as mentioned by Koran [27].

Our results also provided insights on the prevalence of various obsessions and compulsions among Thai OCD patients. The most common symptom reported in this study was compulsively checking things (74.5%). There were other two symptoms that were endorsed by over 50% of the sample: unnecessary rereading or rewriting; repeatedly asking for reassurance, both of which are compulsions. The three most common obsessions were as follows: being obsessively worried about contamination (46.8%); fear of harm coming to the loved ones (46.8%); and worried about fire, burglary, or flooding of the house (42.6%). Our results were different from the study of the English FOCI, which reported that the most common obsession and compulsion were keeping objects in perfect order (60%) and compulsive reassurance seeking (70%), respectively. For other studies, Matsunaga et al. [28] reported contamination obsessions (48%) and cleaning/washing and checking compulsions (47%) were the most common symptoms in Japanese patients, whereas Pinto et al. [26] reported symmetry obsessions (25.8%), contamination obsessions (22.7%), miscellaneous compulsions (21.8%), and checking compulsions (21%) as the most common symptoms in their samples. The different prevalence of obsessive and compulsive symptoms might be due to study designs, measures, and cultural differences, as well as the smaller number of participants in our study.

There were some limitations of the present study that should be acknowledged and would be suggested for the future research. Firstly, this study was run in a university hospital in the capital city of Thailand which might limit some characteristics of samples, such as the relatively high educational level and city life-style of the participants. However, this hospital has a high number of patients and enough OCD patients to collect data for this particular study. Secondly, the number of participants was quite small, which was mostly due to unavailability of some invited OCD patients. Another truly important issue in OCD patients was the feeling of discomfort of the patients to disclose their obsessive-compulsive symptoms [29]. As reported by Hobart et al. [30] the sample size of 20 was stable for reliability and 75% of measurement scales in sample of 40 were stable for validity; the number of 47 participants in this study was therefore considered acceptable to calculate the reliability and validity. Thirdly, the test-retest reliability of the FOCI-T was not assessed because most of the participants attended their follow-up appointment at least one month afterwards, which is too long a hiatus to assess this type of reliability. Other psychometric properties, such as receiver operating characteristics (ROC) to use the FOCI as a screening tool and sensitivity to treatment, were not examined in this study. No study of ROC analyses has been reported yet [31]. This could point to a path for future studies.

## 6. Conclusion

This study has given sufficient evidence of the adequate reliability and validity of the Thai version of the FOCI. It can be a truly useful self-report measure to assess obsessive-compulsive symptoms and severity of OCD patients in clinical settings.

## Figures and Tables

**Table 1:** The frequencies of the Symptom Checklist items and the means; standard deviations; item-total correlations; and factor loadings of the severity items of the FOCI-T.

Item number	Description			% yes	
*Total scale *					
Bothered by thoughts/images such as…					
1	Contamination or acquiring a serious illness			46.8	
2	Keeping objects in perfect order			38.3	
3	Images of death or other horrible events			17.0	
4	Unacceptable religious or sexual thoughts			34.0	
Worried about terrible things, such as…					
5	Fire, burglary, or flooding of the house			42.6	
6	Accidentally hitting a pedestrian with your car			25.5	
7	Spreading an illness			12.8	
8	Losing something valuable			38.3	
9	Harm coming to a loved one			46.8	
10	Acting on an unwanted urge or impulse			23.4	
Driven to perform some acts again like…					
11	Ritualized washing, cleaning, or grooming			44.7	
12	Checking things (e.g., light switches)			74.5	
13	Counting, arranging, and evening-up behaviors			36.2	
14	Collecting useless objects			42.6	
15	Repeating routine actions			31.9	
16	Needing to touch objects or people			12.8	
17	Unnecessary rereading or rewriting			55.3	
18	Examining your body for signs of illness			23.4	
19	Avoiding colors, numbers, or names			21.3	
20	Repeatedly asking for reassurance			63.8	

Severity scale		M (SD)	Corrected item-total correlation	Alpha if item deleted	Factor loadings

21	How much *time* by these thoughts or behaviors	1.93 (1.04)	0.73	0.91	0.83
22	How much *distress* do they cause	1.98 (1.09)	0.90	0.87	0.95
23	How hard is it to *control* them	1.98 (0.91)	0.76	0.90	0.85
24	How much do they cause *avoid*ance behavior	1.61 (1.14)	0.70	0.91	0.81
25	How much do they *interfere* with life	1.67 (1.10)	0.84	0.88	0.91
	Total score	**9.00 (4.67)**			

**Table 2:** Correlations between the FOCI-T and other measures of OCD, depression, and quality of life.

	(1)	(2)	(3)	(4)	(5)	(6)	(7)	(8)	(9)	(10)
(1) FOCI-T Symptom Checklist	**.86**	*.60 *	*.61 *	*.46 *	*.57 *	*.32 *	*−.28 *	*−.31 *	*.49 *	*.52 *
(2) FOCI-T Severity	.53^**^	**.92**	*.93 *	*.90 *	*.97 *	*.76 *	*−.64 *	*−.57 *	*.41 *	*.69 *
(3) Y-BOCS-II Obsession	.54^**^	.86^**^	**.92**	*.79 *	*1.01 *	*.61 *	*−.50 *	*−.50 *	*.47 *	*.63 *
(4) Y-BOCS-II Compulsion	.40^**^	.81^**^	.71^**^	**.88**	*1.01 *	*.69 *	*−.68 *	*−.39 *	*.21 *	*.43 *
(5) Y-BOCS-II Total	.51^**^	.90^**^	.93^**^	.91^**^	**.93**	*.70 *	*−.63 *	*−.48 *	*.37 *	*.57 *
(6) Clinical global Impression scale	.32^*^	.76^**^	.61^**^	.69^**^	.70^**^	**NA**	*−.86 *	*−.28 *	*.23 *	*.37 *
(7) Global Assessment of Functioning	−.28	−.64^**^	−.50^**^	−.68^**^	−.63^**^	−.86^**^	**NA**	*.24 *	*−.11 *	*−.21 *
(8) Pictorial Thai Quality of Life	−.26	−.50^**^	−.44^**^	−.33^*^	−.42^**^	−.28	.24	**.83**	*−.68 *	*−.82 *
(9) Hamilton Rating Scale for Depression	.42^**^	.36^*^	.41^**^	.18	.33^*^	.23	−.11	−.57^**^	**.84**	*.70 *
(10) The nine-item Patient Health Questionnaire	.45^**^	.62^**^	.57^**^	.38^**^	.52^**^	.37^**^	−.21	−.70^**^	.60^**^	**.88**

Min	0	0	0	0	0	1	45	12	0	0
Max	18	19	21	21	39	5	90	63	29	27
Mean	7.32	9.00	10.00	9.09	19.09	3.59	62.98	35.30	6.19	8.38
SD	4.70	4.67	5.94	5.25	10.34	.99	9.15	11.97	6.06	6.22

Note: OCD: obsessive-compulsive disorder; FOCI-T: the Florida Obsessive-Compulsive Inventory; Y-BOCS-II: Yale-Brown Obsessive-Compulsive Scale; NA: not applicable. The upper part (italic font) represents corrected correlations and the reliabilities are in the diagonal (bold).

^*^ *P* < 0.05, ^**^ *P* < 0.01.
